# The Association Between Locus of Control and Psychopathology: A Cross-Cohort Comparison Between a UK (Avon Longitudinal Study of Parents and Children) and a Japanese (Tokyo Teen Cohort) Cohort

**DOI:** 10.3389/fpsyg.2021.600941

**Published:** 2021-04-21

**Authors:** Sarah Sullivan, Syudo Yamasaki, Shuntaro Ando, Kaori Endo, Kiyoto Kasai, Iryna Culpin, Christina Dardani, Stanley Zammit, Atsushi Nishida

**Affiliations:** ^1^Centre for Academic Mental Health, Population Health Sciences, Bristol Medical School, University of Bristol, Bristol, United Kingdom; ^2^Unit for Mental Health Promotion, Research Centre for Social Science & Medicine, Tokyo Metropolitan Institute of Medical Science, Tokyo, Japan; ^3^Department of Neuropsychiatry, Graduate School of Medicine, The University of Tokyo, Tokyo, Japan; ^4^Institute of Psychological Medicine and Clinical Neurosciences, Cardiff University School of Medicine, University of Cardiff, Cardiff, United Kingdom

**Keywords:** psychotic-like experiences, locus of control, externality, ALSPAC, depression

## Abstract

**Background:** An external locus of control (externality) is associated with poorer psychopathology in individualist cultures, but associations are reported to be weaker in collectivist cultures where an external style is less maladaptive. We investigated the prospective association between externality and psychotic-like experiences (PLE) and depressive symptoms (DS) and compared the strength of associations between a UK and a Japanese cohort.

**Method:** Cross-cultural cohort study of a UK (Avon Longitudinal Study of Parents and Children) and a Japanese cohort (Tokyo Teen Cohort). Externality was assessed using the Children's Nowicki and Strickland Internal, External Scale and DS using the Short Moods and Feelings Questionnaire in both cohorts, PLE were assessed with the Psychosis-Like Experiences Questionnaire (ALSPAC), and the Adolescent Psychotic-Like Symptom Screener (TTC). Associations were investigated using multivariable regression models and bivariate regression models to compare the strength of associations.

**Results:** Mean externality in both childhood and adolescence was higher in ALSPAC than in the TTC. Childhood externality was associated with PLE in late childhood and adolescence in both cohorts and adolescent externality was associated with PLE in young adulthood in the ALSPAC cohort. There was a more mixed pattern of association between externality and DS scores. There was little evidence of any differences in the strength of associations between externality and different psychopathologies, or between cohorts. In ALSPAC adolescent externality and early adult psychopathology were more strongly associated than childhood externality and adolescent and early adult psychopathology. There was no evidence that change in externality between childhood and adolescence was associated with new onset PLE or DS in early adulthood.

**Conclusion:** An external locus of control is associated with poor mental health regardless of cultural context.

## Background

The concept of “locus of control” was first defined by Rotter (1966), where he described it as “a generalized problem-solving expectancy functioning within social theory” (Nowicki et al., 2018a). Locus of control (LoC) is a term used to describe the extent to which a person believes that they have control over events and outcomes, in other words whether they have “agency” (Nowicki et al., 2018a). Its role in the link between negative events and psychopathology has been reported both as a moderator (i.e., changing the strength and nature of the relationship) and a mediator (mechanism that explains the relationship) (Liu et al., 2000; Culpin et al., 2015). LoC style is not innate but learnt through reinforcement and social interaction (Rotter, 1966). For this reason the meaning given to LoC is considered to be culturally influenced (Cheng et al., 2013). For instance, in individualist societies (such as the US and UK) where achievement of one's own goals is considered paramount, an externalized LoC where agency is lacking, has negative connotations (Markus and Kitayama, 1999). However, in more collectivist societies (such as many South East Asian communities) achievement of the goals of the group are considered to be more important than achievement of personal goals, and one's identity is constructed via the group rather than the individual. There is evidence that mean externality scores are higher in collectivist societies (Cheng et al., 2013) and members of societies with collectivist cultures are therefore more tolerant of external control (Markus and Kitayama, 1999).

To have an externalized LoC is to believe that one has little personal control and that outcomes and events can be attributed to outside forces such as other people and chance (Rotter, 1966). Conversely, to have an internalized LoC is to believe that one has control over events and outcomes (Rotter, 1966; Pagnini et al., 2016). An internalized LoC has been shown to be adaptive and associated with higher levels of well-being (Chorpita and Barlow, 1998), perhaps because it can be used as a coping strategy (Cheng et al., 2013). An externalized LoC reduces propensity to engage in problem-solving activities (Cheng et al., 2013) and has been linked to depression, anxiety and psychotic like experiences (PLE) in adults and adolescents (Sullivan et al., 2017). It is important to establish whether a more external LoC has a causal effect on psychopathology (Kurtovic et al., 2018) particularly as, there is evidence (Jarrett et al., 2007) that LoC style is modifiable with appropriate psychological therapy.

There are some aspects of the relationship between LoC and psychopathology, which are currently under-explored. Firstly, whilst most previous research has examined the link between LoC style (internal or external) and depression and anxiety, less work has focussed on the association with PLE. It is important to investigate this link because LoC has a major influence on constructions of reality, the concept of self, and representations about others, all of which are impaired in clinical psychosis and in people with PLE in general population samples (Bentall et al., 1994; Donohoe et al., 2008; Langdon et al., 2010). Many of the positive symptoms of psychosis, such as hallucinations and delusions are associated with a loss of contact with reality and a blurring of the boundary between the self and the outside world (Cicero et al., 2016). For example, auditory hallucinations are hypothesized to arise as a result of misattributing internal thoughts or memories as an external “voice” emanating from another person. On the other hand, externality is also associated with feelings of helplessness arising from a belief of lack of control, which is a common feature of depression (Chorpita, 2001).

Secondly, very little work has compared whether the association between LoC and PLE is of a similar magnitude to that between LoC and depressive symptoms (DS). This is important because PLE and DS frequently co-occur (Sullivan et al., 2014) and therefore many risk factors are common to both. It is important to unpick the complex associations between risk factors for PLE and comorbid DS to better inform the etiology and mechanism of PLE and DS, help to detect those at specific risk and inform interventions that may help to reduce the incidence of psychosis and depression. A direct comparison of the effect size between externality and PLE and DS using appropriate statistical methods that allow for overlap in the outcomes, will facilitate an estimate of whether externality is a more important risk factor for PLE or DS.

Thirdly, less research has been conducted on the link between LoC style and psychopathology in children and adolescents than in adults, There is evidence (Nowicki et al., 2018b) that externality reduces with maturity in healthy populations. It is important to clarify whether the same relationships between LoC style exist in children and adolescents as in adults because most mental health problems have their origins in late childhood and early adolescence (Kessler et al., 2007) and it is at this stage of life when early intervention is likely to have the best long-term result.

Fourthly, most research has been carried out in the UK and US where the culture is more individualist, which does not elucidate the relationship in populations with collectivist cultures (Cheng et al., 2013; Moreira et al., 2020). There is some cross-sectional evidence that people in collectivist counties report higher external LoC scores and that LoC scores show weaker associations with psychopathology (Cheng et al., 2013). It is important to compare the relationship between externality and psychopathology across cultures because the importance of externality in the etiology of these mental health problems may not be universal, but culturally dependent.

Lastly, most previous research into externality and mental health problems has been cross-sectional and cannot provide insight into the temporal sequence of LoC style and psychopathology. The authors are only aware of relatively few previous longitudinal studies (Frenkel et al., 1995; Harrow et al., 2009; Thompson et al., 2011; Wiersma et al., 2011; Culpin et al., 2015). Two (Thompson et al., 2011; Culpin et al., 2015) were conducted in one of the same cohorts as the study described here, but one (Culpin et al., 2015) investigated the association of LoC as a mediator between adverse events and depression and the other (Culpin et al., 2015) the association between childhood LoC and PLE at age 12. A further study (Wiersma et al., 2011) only investigated the association between LoC and depression and the remaining two had small (*n* = 89 and 128) sample sizes.

Given the problem of inferring causality in observational research, cross-cultural studies provide especially valuable evidence as these can help determine whether residual confounding is present. Cohorts in countries which are culturally and ethnically diverse are likely to have different confounding variable structures. If the same, or similar, findings are established in two cohorts with different confounding structures, confidence that associations are causal is increased (Richmond et al., 2014). This method has been used in epidemiological studies examining whether the effect of maternal age on birthweight and gestational age is causal or due to confounding (Restrepo-Mendez et al., 2015). Cross cultural cohort comparisons are further strengthened if the instruments used to measure risk factors and outcomes are the same or similar (Richmond et al., 2014).

Little research has been carried out into individual change in externality over time in children except by Nowicki et al. (2018a). Children with a normal developmental course should become more internal over time as they acquire more control over their surroundings as they mature. Hypothetically therefore, those with stable or increasing externality may have a different developmental trajectory from their peers.

There is some recent unpublished evidence in a UK general population cohort that an externalized LoC during childhood and adolescence is a risk factor for new onset of PLE between late adolescence and early adulthood. It would be informative to investigate whether an increase in externality over time is also associated with new reports of PLE.

This study aims to investigate and compare the longitudinal associations between externality and both PLE and DS in children and adolescents in a UK general population cohort (Avon Longitudinal Study of Parents and Children, ALSPAC) and a Japanese general population cohort (Tokyo Teen Cohort, TTC).

We predicted that: (1) Externality in childhood and adolescence would be associated with PLE and DS in adolescence and early adulthood in both cohorts, but that the association between externality in adolescence and psychopathology in early adulthood would be stronger than that between childhood externality and psychopathology in early adolescence; (2) The association between externality and PLE would be stronger than that between externality and DS in both cohorts; (3) The associations between externality and PLE and DS would be stronger in the ALSPAC cohort compared to the TTC cohort; (4) An increase in externality between childhood and adolescence would be associated with new onset PLE and DS in early adulthood.

## Methods

### Samples

The Avon Longitudinal Study of Parents and Children (ALSPAC) was designed to determine the environmental and genetic factors that are associated with the health and development of the study offspring (Boyd et al., 2013; Fraser et al., 2013; Northstone et al., 2019). ALSPAC recruited 14,541 pregnant women residing in Avon, an area of south-west England, with expected dates of delivery between 1st April 1991 and 31st December 1992. Of these initial pregnancies, there was a total of 14,676 fetuses, resulting in 14, 062 live births, 13, 988 of whom were alive at 1 year of age. Data were collected at various time-points using self-completion questionnaires, biological samples, hands-on measurements, teacher reports, and linkage to other data sets. Please note that the study website contains details of all available data through a fully searchable data dictionary http://www.bristol.ac.uk/alspac/researchers/our-data/.

The self-report data of the cohort from age 22 were collected using REDCap (2020).

Tokyo Teen Cohort (TTC), http://ttcp.umin.jp/index.html, is a longitudinal survey of children and adolescents. Details of the TTC are described elsewhere (Yamasaki et al., 2019). A sample of 3,171 households with children aged 10 years who were born between September 2002 and August 2004 were randomly chosen from residential registries of three municipalities (Chofu, Mitaka, and Setagaya) in Tokyo, Japan. When these children were 12 years old 3007 households participated in the second wave of the study (follow-up rate: 94.8%). Trained interviewers visited the participants' home twice during each wave. On the first visit, the interviewers obtained written informed consent from the adolescents' primary parent and asked them to complete a set of questionnaires. On the second visit, they conducted assessments including semi-structured interviews and neuropsychological tests and measured anthropometric data. When the participants were 14 years old 2,667 households (84.1%) participated in the third wave of data collection when similar assessments were conducted to the first two waves.

**Figure 1:**
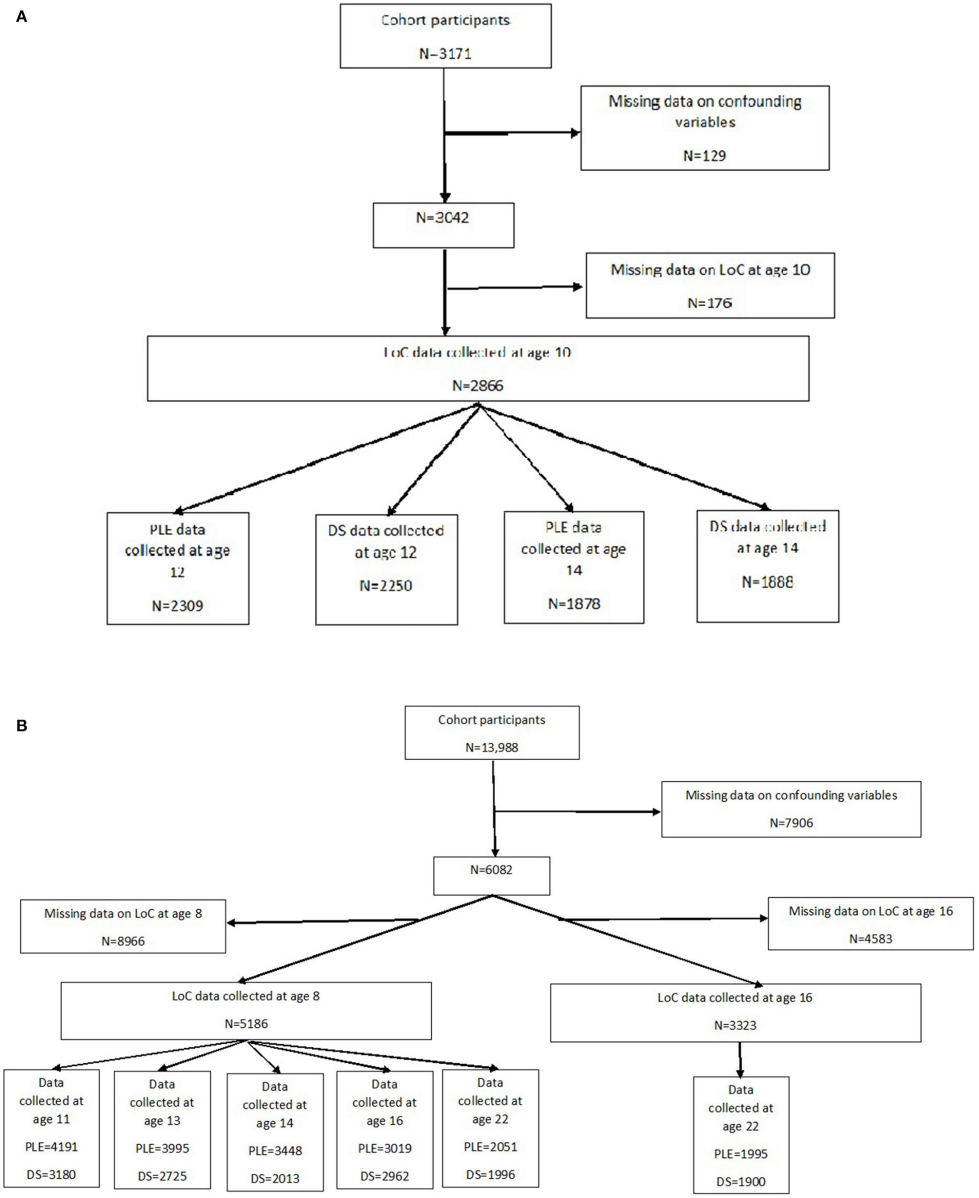
**(A)** Flowchart of study subsample-Tokyo teen cohort. **(B)** Flowchart of study subsample-ALSPAC.

For the purposes of this investigation in both cohorts we used a subset who had provided data on LoC, PLE, DS and relevant confounding variables at various timepoints (Figures 1A,B).

### Ethics

Ethic approval for this study was obtained from the ALSPAC Ethics and Law Committee (ALEC; IRB00003312) (registered on the Office of Human Research Protections database as U Bristol IRB #1), and the Local Research Ethics Committees. The Committees agreed that consent was implied if questionnaires were returned.

The study protocol of the TTC was approved by the institutional review boards of Tokyo Metropolitan Institute of Medical Science, SOKENDAI (Graduate University for Advanced Studies), and the University of Tokyo.

### Outcome Measures

#### Psychotic-Like Experiences

##### Tokyo Teen Cohort

Assessed using the self-report questionnaire—Adolescent Psychotic-Like Symptom Screener (APSS) (Kelleher et al., 2011) at 12 and 14 years. The APSS is a 7-item measure consisting of 4 questions from the Diagnostic Interval Schedule for Children (Shaffer et al., 2000) (“have you ever heard voices that no one else can hear?,” “have you ever thought that people are following or spying on you?,” “have other people ever read you mind?,” “have you ever had special messages sent to you through TV or radio?”) and 3 additional questions on, visual hallucinations, delusions of control and grandiosity. At age 12 only 5 of the questions (auditory and visual hallucinations, paranoia, and delusions of others reading your mind and reference) were asked. At 14 years all seven questions were asked, plus two extra questions on body dysmorphia and delusion of reading others' minds. For each question the respondent was asked about their lifetime experience and there were three possible responses: yes definitely, yes maybe and no. At 14 years only, questions were also asked about distress (fine, a little distressing, very distressing) and frequency (if definite: only once and twice or more). The APSS has good sensitivity and specificity in identifying young people who had PLE that were subsequently identified by clinical interview (Kelleher et al., 2011). For the purposes of analysis a binary variable was defined at each timepoint consisting of definite PLE vs. maybe/no PLE.

##### ALSPAC

Assessed using the child/adolescent/adult completed PLIKS-Q questionnaire at 5 timepoints: 11.5, 13, 14, 16.5, and 22 years. The PLIKS-Q is a specially designed questionnaire, based on the PLIKS-I interview (Horwood et al., 2008), asking about lifetime occurences, level of conviction (definitely or maybe), past year frequency (“none,” “ < once per month,” “≥monthly”) and the attribution of the PLE. The PLE enquired about were visual, and auditory hallucinations, beliefs about being spied on, beliefs that others were using special powers to read their thoughts, beliefs that they were being sent special messages, or beliefs that some special power was controlling them. At 11.5 years 7 questions were asked, at 13, 14, and 16.5 years 11 questions were asked and at 22 years 3 questions were asked about the number of different PLE.

The data at each timepoint, in each cohort, were coded as binary (present if the respondent endorsed a PLE as having ever definitely occurred and absent if it was endorsed as only maybe occurring or not present).

#### Severity of PLE

A 5-category variable indexing the severity of PLE was created using information on frequency and distress caused by PLE. The categories were none, maybe, definite but not distressing or frequent, definite, and distressing (quite or very) OR frequent (at least monthly), definite and distressing (quite or very) AND frequent (at least monthly). This variable was defined in ALSPAC at 14, 16, and 22 years and in TTC at 14 years. The frequency categories collected by the TTC at 14 years were different from ALSPAC so the highest frequency category (at least twice) was used to allow a more direct comparison between cohorts.

#### Depressive Symptom Scores

DS scores were collected in both the ALSPAC and Tokyo Teen Cohort using the Shortened Moods and Feelings Questionnaire (Angold et al., 1995) at 10, 12, and 14 years (TTC) and 11, 13, 14, 16, and 22 years (ALSPAC). Thirteen questions were asked about how the participant had been feeling over the past 2 weeks with possible responses of not true (score = 0), sometimes true (score = 1), and always true (score = 2). For the purposes of analysis the total DS score was used (range 0–26). Higher scores indicated worse DS. In both cohorts the questions were self-completed by participants.

For the bivariate analysis described below a binary measure of depression was derived using a cut-off score of ≥11 to allow statistical modeling alongside the binary PLE variable. This cut off has been shown to have a high specificity and sensitivity (Thapar and McGuffin, 1998) and has been previously applied in community samples (Copeland et al., 2009).

#### New Onset PLE and Depression

New onset PLE in the ALSPAC cohort was defined as having PLE occurring at least once since age 20 at the age 22 assessment but not ever in the previous assessments. New onset depression (see binary variable definition above) was defined as meeting the criteria for depression in the 2 week period prior to the age 22 assessment, but not in the previous assessments.

This analysis was not carried out in the TTC because LoC was only measured at two timepoints (10 and 14 years) and there were no further measures of PLE and DS after age 14 years.

### Exposure Measure

#### Locus of Control

The Children's Nowicki and Strickland Internal, External Scale (CNSIE) (Nowicki and Strickland, 1973) originated from an administration of the 40-item test to a sample of 120 8 year olds, following which the 12 items with the best item-total correlation were chosen for inclusion in the final form administered to both cohorts. With regard to discriminative validity, the CNSIE showed little evidence of associations with social desirability (Nowicki and Strickland, 1973) and with regard to construct validity, there was evidence of moderate relations between the CNSIE and other measures of LoC (e.g., Intellectual Achievement Responsibility and Bailer-Cromwell scales) (Nowicki and Strickland, 1973). There is also evidence of association between internality and higher academic achievement (Nowicki and Strickland, 1973).

In ALSPAC, LoC was measured in participants at 8 and 16 years of age. When cohort participants were 8 years old the questions were read aloud to participants by an interviewer to eliminate differences in reading ability. At 16 years of age the questionnaires were self-completed without any assistance. In the TTC LoC was assessed at 10 and 14 years of age. The CNSIE was self-completed by participants. Externality score in these measures was calculated as the number of external responses. Higher scores represented a more externalized style.

We also calculated a variable for change in LoC. For the ALSPAC cohort we subtracted the scores at 8 from the scores at 16 years. For the Tokyo Teen cohort we subtracted the scores at 10 years from those at 14 years. Negative scores represented a decrease in externality (an increase in internality) over time, whereas positive scores represented an increase in externality (a decrease in internality).

### Confounders

The choice of covariates was informed by the literature. Gender, socio-economic status (highest parental education, home ownership status, income, mother's marital status) (see Supplementary Table A for details) and IQ (measured using the Weschler Intelligence Score for Children) were selected. As far as possible similar variables were chosen in each cohort (see Supplementary Table B for cross-cohort comparison). In ALSPAC the covariates were measured at the child's birth, except for IQ which was assessed at age 8. In TTC the covariates were measured at baseline when the child was 10 years of age. To define family income the variable was dichotomised at the top 30% of income in both cohorts to remove the effect of currency differences. In ALSPAC mothers were asked if they were married to the child's father when the child was born whereas in the TTC mothers were asked if they lived with the child's father when the child was born.

### Statistical Analysis

For all analyses, the same complete cases sample was used for both the univariable and multivariable models. The sample size varied for each model depending on the timepoint of the exposure and outcome investigated. Univariable and multivariable logistic regression was used to investigate the association between externality and PLE (ALSPAC externality at 8 years and PLE at 11, 13, 14, 16, and 22 years: TTC externality at 10 years and PLE at 12 and 14 years) and univariable and multivariable linear regression to investigate the association between externality and DS scores (ALSPAC externality at 8 years and DS scores at 11, 13, 14, 16, and 22 years: TTC externality at 10 years and DS scores at 12 and 14 years). Univariable and multivariable logistic regression were used to investigate the association between change in externality and new onset PLEs and depression (using the ≥11 SMFQ cut off). We used a bivariate probit analysis to jointly model the outcomes of PLE and depression (using the ≥11 SMFQ cut off) to compare the strength of association between externality and PLE and depression whilst allowing for overlap between outcomes. We used a Wald test to provide evidence against the null hypothesis that the estimates of association were equal for each outcome. A small Wald Test *p*-value would indicate strong evidence against the null hypothesis. Probits were converted to odds ratios for ease of interpretation by exponentiating and then multiplying by 1.6 (Amemiya, 1981). An ordinal logistic regression analysis was used to analyse the association between externality and severity of PLE.

## Results

### Cross-Cohort Descriptive Statistics (Table 1)

Both sexes were almost equally represented in each cohort although the proportion of males in the TTC was higher. Mean IQ and the proportion of parents owning their home was similar across cohorts, although the proportion of parents who had completed higher education was much higher in the TTC compared to ALSPAC.

**Table 1:** Description of all variables across cohorts.

**Measure**	**Scale**	**Analysis units**	**Sample *n***	***n* (%)/mean (SD) exposed**	**Scale**	**Analysis units**	**Sample *n***	***n* (%) exposed**
	**ALSPAC**				**Tokyo teen cohort**			
**Confounders**								
Sex	Binary	Female	7,156	3,573 (49.9%)	Binary	Female	3,171	1,487 (46.9%)
IQ	WISC range (74–151)		7,156	Mean 102.3 (SD 21.6)	WISC range (50.9–144.3)		3,168	Mean 107.7 (SD 14.1)
Homeownership status of mother at child's birth	Binary	Mortgaged or owned	12,993	9,529 (70.8%)	Binary	Homeownership of parent's asked when child 10 years old	3,164	2,212 (69.9%)
Marital Status of mother at child's birth	Binary	Married at child's birth	13,053	9,780 (75.0%)	Identity of partner from child's perspective at 10 years old	Biological father-asked when child 10 years old	3,171	3,121 (98.4%)
Highest maternal education level at child's birth	Binary	Higher education at child's birth (A levels/degree)	12,383	4,378 (33.4%)	Binary	At least one parent completed higher education asked when child age 10 years old	3,170	2,257 (71.2%)
Average household income when child 33 and 47 months	Binary	Top 30%			Binary	Household income when child 10 years old–top 30% of cohort over previous year	3,046	917 (30.11%)
**Risk factors**								
Locus of control @ 8 (0-12)	CNSIE		6,101	Mean 6.00 (SD 2.07)				
Locus of control @ 10 (0–12)					CNSIE		2,983	Mean 3.99 (SD 1.92)
Locus of control @ 14 (0–12)							2,399	Mean 3.63 (SD 1.9)
Locus of control @ 16 (0–12)			4,772	Mean 4.20 (SD 2.12)				
**Outcomes**								
Depression @ 11 years	SMFQ	Depressed ≥score of 11	5,716	516 (9.03%)				
Depression @ 12 years					SMFQ	Depressed ≥score of 11	2,479	233 (9.4%)
Depression @ 13 years			4,248	770 (18.1%)				
	**ALSPAC**				**Tokyo teen cohort**			
Depression @ 14 years			3,187	585 (18.4%)			2,070	169 (8.2%)
Depression @ 16 years			4,773	885 (17.9%)				
Depression @ 22 years			3,149	569 (18.1%)				
New onset depression @ 22		Depressed ≥score of 11 over previous 2 weeks but not at previous timepoints	1,319	53 (4.0%)				
PLE ever @ 11	PLIKSQ Binary	Definite vs. maybe/no	7,173	1,860 (25.9%)				
PLE ever @ 12					APSS binary	Definite vs. maybe/no	2,539	458 (18.0%)
PLE ever @ 13			6,758	1,116 (16.5%)				
PLE ever @ 14			5,719	741 (13.0%)			2,062	345 (16.7%)
PLE ever @ 16			4,884	648 (13.3%)				
PLE ever @ 22			3,242	196 (6.1%)				
New onset PLE @ 22		More than once since 20th birthday but no previous reports at earlier ages	2,158	35 (1.6%)				
Severity of PLE @ 14	PLIKSQ 5 category	None	5,718	3,330 (58.2%)	APSS 5 category	None	2,061	1,519 (73.7%)
		Maybe		1,712 (29.9%)		Maybe		326 (15.8%)
		Definite but not distressing or frequent		287 (5.0%)		Definite but not distressing or frequent		29 (1.4%)
		Definite and distressing OR frequent		302 (5.3%)		Definite and distressing OR frequent		103 (5.0%)
		Definite and distressing AND frequent		87 (1.5%)		Definite and distressing AND frequent		84 (4.1%)
Severity of PLE @ 16	PLIKSQ 5 category	None	4,884	3,260 (66.8%)				
		Maybe		1,042 (21.3%)				
	**ALSPAC**				**Tokyo teen cohort**			
		Definite but not distressing or frequent		276 (5.7%)				
		Definite and distressing OR frequent		247 (5.1%)				
		Definite and distressing AND frequent		59 (1.2%)				
Severity of PLE @ 22	PLIKSQ 5 category	None	3,242	2,571 (79.3%)				
		Maybe		475 (14.7%)				
		Definite but not distressing or frequent		100 (301%)				
		Definite and distressing OR frequent		71 (2.2%)				
		Definite and distressing AND frequent		25 (0.77%)				

In the TTC the mean externality score was 3.99 (SD 1.92) at age 10 and at age 14 it was 3.63 (SD 1.92). In the ALSPAC cohort, the mean externality score at age 8 was 6.00 (SD 2.07) and at 16 years it was 4.18 (SD 2.11). In both cohorts the mean externality score decreased with age. The mean decrease per year in ALSPAC was 0.21 per year and in the TTC it was 0.08 per year. This finding should be interpreted cautiously because it assumes that the change over time is linear across all ages and in both cohorts, which may not be the case.

The proportion of those depressed (SMFQ score ≥11) was very similar in ALSPAC at 11 years and TTC at 12 years but higher in ALSPAC at 14 years compared with the TTC at the same age. In ALSPAC the proportion defined as depressed increased between 11 and 13 and remained higher than the TTC, whereas in the TTC the proportion defined as depressed decreased between 12 and 14 years.

The proportion with PLE was lower in the TTC compared with ALSPAC at 11 and 12 years but more comparable at 14 years. In both cohorts the proportion reporting PLE decreased over time. The proportions of those in the most severe PLE category (i.e., definite, frequent, AND distressing) was higher in the TTC than ALSPAC at 14 years, although there was a higher proportion in TTC who had not reported any PLE. See Supplementary Table C.

### Cross Cohort Comparison of Association Between Externality and PLE

Externality scores were normally distributed in both cohorts. Externality in childhood and adolescence was associated with PLE at all time points and adjusting for covariates had little effect on the estimates. The strength of associations reduced as the length of time between the LoC measure and outcome increased. The strength of association between externality and PLE ranged from a 5% to a 26% increase in odds of PLE for each point increase in externality score. The association was stronger in the TTC compared to ALSPAC but all confidence intervals overlapped. See Table 2A.

**Table 2A:** Multivariable^*^ associations between externality and PLE.

**Timepoint of PLEs data collection years**	**11**			**12**			**13**			**14**			**16**			**22**		
	**OR**	**95% CI**	***p***	**OR**	**95% CI**	***p***	**OR**	**95% CI**	***p***	**OR**	**95% CI**	***p***	**OR**	**95% CI**	***p***	**OR**	**95% CI**	***p***
**ALSPAC**																		
Sample size n	4,190						3,995			3,48			3,019			2,051/1,955		
Externality @ 8	1.06	1.02–1.10	0.002				1.08	1.03–1.13	≤0.0001	1.07	1.01–1.11	0.047	1.06	1.00–1.12	0.062	1.04	0.94–1.14	0.471
Externality @ 16																1.26	1.15-1.38	≤0.0001
**TTC**																		
Sample size n				2,309						1,878								
Externality @ 10				1.12	1.06–1.19	≤0.0001				1.11	1.04–1.18	0.002						

*Complete cases. Univariable results in Supplementary Table E*.

* *Adjusted for sex, IQ at age 8, home ownership status, parental marital status, parental educational level, family income*.

There was no evidence that change in externality between 8 and 16 years in the ALSPAC cohort was associated with new onset of PLE in early adulthood. See **Table 3**.

In both cohorts there was strong evidence that the association between higher externality scores and PLE becomes stronger with increasing severity of experiences. In the TTC the association was 2.4 times stronger between the least severe (maybe) and the most severe (distressing and frequent) PLE category, whereas in ALSPAC the difference ranged from nearly 3 to 6 times stronger (Table 4).

### Cross Cohort Comparison of Association Between Externality and DS

There was evidence of an association between externality score and DS score in both cohorts at all timepoints, although at the earlier timepoint the association in the TTC (externality at age 10 and DS at age 12) was stronger than in ALSPAC (externality at age 8 and DS at age 11) whereas at the later timepoint (TTC: externality at age 10 and DS at age 14 and ALSPAC: externality at age 8 and DS at age 14) the strength was similar. Predictably, as the time-period between measurement of externality and DS scores increased the estimate of association weakened. In ALSPAC the association between externality in mid adolescence (16 years of age) and DS in young adulthood was about 4 times stronger than that between externality in childhood (8 years of age) and DS in mid adolescence (at 16 years of age) even though the time-period of measurement between risk factor and outcome was the same (6 years; Table 2B).

**Table 2B:** Multivariable^*^ associations between externality and sFMQ score (0–26).

**Timepoint of sMFQ data collection (years)**	**11**			**12**			**13**			**14**			**16**			**22**		
	***β***	**95% CI**	***p***	***β***	**95% CI**	***p***	***β***	**95% CI**	***p***	***β***	**95% CI**	***p***	***β***	**95% CI**	***p***	***β***	**95% CI**	***p***
**ALSPAC**																		
Sample size n	3,810						2,725			2,150			3,156			1,996/1,900		
Externality @ 8	0.14	0.07–0.21	≤0.0001				0.080	−0.02–0.17	0.100	0.13	0.004–0.225	0.43	0.13	0.03–0.22	≤0.0001	0.11	−0.01–0.230	0.071
Externality @ 16																0.58	0.46-0.70	≤0.0001
**TTC**																		
Sample size n				2,250						1,888								
Externality @ 10				0.34	0.24–0.44	≤0.0001				0.20	0.08–0.31	0.001						

*Complete cases. Univariable results in Supplementary Table F*.

* *Adjusted for sex, IQ at age 8, home ownership status, parental marital status, parental educational level, family income*.

There was no evidence of an association between change in externality between 8 and 16 years in the ALSPAC cohort and new onset of depression at 22 years. See Table 3.

**Table 3:** Univariable and Multivariable^*^ association between change in externality between 8 and 16 (−9–8) and new onset PLE and depression between 16 and 22 (reported at 22 but not at 16, 14, 13, or 11).

	***n***	**Unadjusted OR**	**95% CI**	***p***	**Adjusted OR**	**95% CI**	***p***
New onset PLE @ 22	1,418	1.05	0.90, 1.23	0.514	1.02	0.87, 1.20	0.769
New onset depression @ 22	947	1.02	0.90, 1.15	0.811	0.970	0.849, 1.108	0.657

*Complete cases (ALSPAC only)*.

* *Adjusted for sex, IQ at age 8, home ownership status, parental marital status, parental educational level, family income*.

**Table 4:** Association between severity of PLE (5 categories) and externality.

**Timepoint of PLE data collection (years)**		**14**				**16**				**22**			
		**Unadjusted**		Adjusted^*^		**Unadjusted**		Adjusted^*^		**Unadjusted**		Adjusted^*^	
		**OR**	**95% CI**	**OR**	**95% CI**	**OR**	**95% CI**	**OR**	**95% CI**	**OR**	**95% CI**	**OR**	**95% CI**
**ALSPAC**													
Sample size n		3,704				3,221				2,199/2,066			
Externality @ 8	None (ref cat)	1		1		1		1		1		1	
	maybe	0.76	(0.57, 0.96)	1.23	(1.00, 1.45)	1.23	(1.00, 1.45)	2.02	(1.34, 2.70)	1.90	(1.58, 2.23)	1.45	(0.48, 2.42)
	Definite but not distressing or frequent	2.44	(2.22, 2.65)	2.58	(2.34, 2.83)	2.58	(2.34, 2.83)	3.39	(2.71, 4.07)	3.20	(2.85, 3.55)	2.75	(1.77, 3.73)
	Definite and distressing OR frequent	3.06	(2.83, 3.29)	3.24	(2.98, 3.50)	3.24	(2.98, 3.50)	4.05	(3.36, 4.74)	3.92	(3.53, 4.31)	3.47	(2.48, 4.47)
	Definite and distressing AND frequent	4.56	(4.24, 4.89)	4.81	(4.43, 5.18)	4.81	(4.43, 5.18)	5.62	(4.88, 6.36)	5.44	(4.84, 6.03)	5.00	(3.91, 6.09)
Sample size *n*		3,221											
Externality @ 16	None (ref cat)									1		1	
	Maybe									2.56	(2.30, 2.83)	2.45	(1.50, 3.40)
	Definite but not distressing or frequent									3.94	(3.62, 4.25)	3.83	(2.86, 4.79)
	Definite and distressing OR frequent									4.60	(4.24, 4.96)	4.49	(3.51, 5.47)
	Definite and distressing AND frequent									6.11	(5.52, 6.69)	6.01	(4.91, 7.09)
**TTC**													
Sample size n	1,877												
Externality @ 10	None (ref cat)	1		1									
	Maybe	1.43	(1.19, 1.66)	1.50	(0.54, 2.45)								
	Definite but not distressing or frequent	2.54	(2.28, 2.80)	2.60	(1.63, 3.56)								
	Definite and distressing OR frequent	2.71	(2.44, 2.97)	2.76	(1.80, 3.73)								
	Definite and distressing AND frequent	3.54	(3.23, 3.85)	3.61	(2.63, 4.59)								

* *Adjusted for sex, IQ at age 8, home ownership status, parental marital status, parental educational level, family income*.

### Comparison of Strength of Association Between Externality and Psychopathology (Table 5)

There was no evidence, in either cohort, of any difference in the strength of association between externality score and PLE compared to that between externality score and depression.

**Table 5:** Main effects (OR and 95% Cis) of externality on depression and PLE.

	**Age**	**Depression**		**PLE**		**Wald test of common effect *p***
		**OR**	**95% CI**	**OR**	**95% CI**	
**ALSPAC**						
Externality@8 years	11	1.04	1.00, 1.09	1.07	1.03, 1.11	0.300
	13	1.06	1.02, 1.11	1.12	1.07, 1.17	0.083
	14	1.10	1.05, 1.16	1.06	1.00, 1.12	0.244
	16	1.07	1.03, 1.11	1.05	1.00, 1.09	0.326
	22	1.04	1.00, 1.09	1.04	0.98, 1.11	0.998
Externality@16 years	22	1.81	1.76, 1.86	1.79	1.72, 1.86	0.623
**TTC**						
Externality@10 years	12	1.12	1.06, 1.19	1.11	1.06, 1.17	0.777
	14	1.10	1.03, 1.18	1.09	1.03, 1.15	0.834

## Discussion

As far as the authors are aware this is the only study to investigate the longitudinal association between externality in childhood and adolescence and PLE and DS in adolescence and early adulthood in a UK (individualist) and a Japanese (collectivist) cultural setting.

As predicted, we found strong evidence of an association between externality and PLE and DS in both cohorts and evidence in the ALSPAC cohort that these associations were stronger between adolescent externality and early adult psychopathology than between childhood and adolescent psychopathology. However, contrary to our prediction there was no evidence that the association between externality and PLE was stronger than that between externality and DS in either cohort or that the associations were stronger in the ALSPAC compared to the TTC cohort. Neither did we find that increasing externality between childhood and adolescence was associated with new onset PLE or DS in the ALSPAC cohort but there was evidence that the association between externality and PLE was stronger as the severity of PLE increased.

As described above and contrary to our hypotheses and the findings of others (Lu et al., 2000) we did not find that mean externality scores were higher in the Japanese cohort nor that the association between externality and psychopathology was weaker when compared with the UK cohort. Others (Kozma and Stones, 1987) have suggested that externality self-report measures may be prone to measurement error because of social desirability. For example, participants from individualist countries may be less likely to endorse externality because they consider it to be an undesirable trait. In contrast, we found ALSPAC participants to have a higher mean externality score than TTC participants.

Our finding of a moderately strong association between externality and DS was similar to that found by others (Cheng et al., 2013; Culpin et al., 2015; Moreira et al., 2020). A meta-analysis (Cheng et al., 2013) found a weaker association between externality and DS for collectivist compared to individualist cultures, which was not replicated in our study.

Our finding of a strong association between externality and PLE reinforces the findings of others (Thompson et al., 2011; Sullivan et al., 2017) using ALSPAC data. One of these studies (Sullivan et al., 2017) found weak evidence of a stronger association between externality at age 8 and PLE compared with depression at age 12 years. This was not replicated in our findings, which might be because a different tool was used to measure PLE. In the Sullivan 2017 study (Sullivan et al., 2017) data from a semi-structured clinical interview was used, whereas in the current work PLE data were collected using a self-report questionnaire. The semi-structured interview method allows the interviewer to probe the answer provided by the respondent using further questions. It could be hypothesized therefore, that there is more measurement error when PLE data are collected by self-report. This measurement error is likely to be non-differential with respect to LoC, and therefore will dilute any association between our exposure and outcomes, and may be why we failed to detect a difference in the strength of association of externality between PLE and depression. This assumes that self-report assessment of DS is more accurate than that of PLE. There is some evidence (Stuart et al., 2014) in favor of this.

Hypothetically, the association between externality and psychopathology should be stronger at later ages because as children mature, they acquire greater control over their environment and themselves and naturally become less externalized. In other words, externality in childhood is less abnormal than during adolescence and hence the associations of externality with PLE and DS might be expected to be weaker in childhood. Although externality decreased with age as expected in both cohorts, it decreased less per year in the TTC than in ALSPAC and in fact externality in ALSPAC remained at a higher level than in the TTC at later ages. In the main, there was little difference in the strength of association between externality and psychopathology at later compared to earlier ages in either cohort.

A possible explanation for the lack of support for our prediction that the association between externality and psychopathology would be weaker in the TTC than that in ALSPAC is the contemporary nature of the TTC data compared to the ALSPAC data. Externality data in ALSPAC was collected ~22 (LoC at age 8) and 14 (LoC at age 16) years ago, whereas the TTC externality data was collected ~6 (LoC at age 10) and 2 (LoC at age 14) years ago. The more recent Japanese data could reflect either a change toward individualism in recent years or that UK culture was less individualist in the early 1990s than it is now. It is not possible to confirm these hypotheses in this study, although there is some recent evidence from a review that modern Japan is a less collectivist society (Takano and Osaka, 2018).

### Strengths and Limitations

A major strength of our work is the cross-cultural comparison in two cohorts which have different confounding structures due to cultural and ethnicity differences (see Supplementary Table B). We observed little evidence of confounding in either cohort, but residual confounding remains a possibility. Because of the differences in confounding structure across cohorts the similarity of the cross-cohort findings suggests that we can be more confident that residual and unmeasured confounding has not materially affected the findings and that our results are consistent with a causal effect of externality on PLE and DS. Cross-cultural cohort comparisons are also an effective way of investigating the effect of selection bias due to loss to follow up (Richmond et al., 2014), particularly if this varies across cohorts. The pattern of missing data (see Supplementary Table D) demonstrates differences in missingness across cohorts but the similarity of the findings suggests that this is not adequately explained by selection bias, further strengthening the inferences that can be made about the causal nature of the observed associations between externality and PLE and DS.

Other strengths of our study include the large sample sizes and the longitudinal nature of the data. The large sample size means we have adequate statistical power to detect relatively small effects, and hence the likelihood that the associations we observe are due to chance is reduced. Furthermore, the prospective study design minimizes the possibility of reverse causation. Another strength is the direct comparison of association strength across cohorts made possible by using the same assessment tools to measure externality and DS and very similar instruments to measure PLE. We also used a statistical test to directly compare the strength of associations between externality and psychopathology, which is rarely reported by other studies.

There are inevitably limitations to our work. ALSPAC and the TTC have suffered from participant attrition, resulting in missing data. Because it is often the participants with more psychological problems that drop out of cohorts, we are likely to have a complete cases sample that is psychologically healthier than those lost to follow up, resulting in selection bias. If those with greater externality are also more likely to drop out, this would likely lead to us under-estimating effects of externality on psychopathology. Previous work carried out in the ALSPAC cohort suggests that using imputed data to account for missingness effects the prevalence but not the strength of associations (Wolke et al., 2009).

We did not have individual measures of individualism or collectivism and there may be considerable individual variation on the individualism-collectivism spectrum within each cohort. There is some evidence that within-cultural variation is larger for collectivist countries that have undergone rapid modernization (Cheng et al., 2011), like Japan. If individual levels of individualism or collectivism are directly related to externality, greater variation on the individualism-collectivism spectrum would change the association between externality and PLE and DS. This problem is also compounded by the fact that in both cohorts the participants were recruited from a specific area of each country. In ALSPAC all participants lived in a small area of the south-west of England, whereas in the TTC all participants were from 3 regions of Tokyo. It is possible that these regions are not representative of the UK or Japan as a whole and vary in their individualism or collectivism from the rest of the country.

We were not able to directly compare across most ages between the cohorts because of differences in data collection timepoints but we were able to compare more generally between periods of life.

As stated above we were only above to estimate the association between externality and self-reported PLE, rather than more accurate data collected using semi-structured interviews, because only self-report data were collected in both cohorts.

## Conclusion

Externality was prospectively associated with later reports of both PLE and DS. The strength of the association did not vary by cultural setting suggesting that belief that one lacks agency over negative events may be universally associated with poorer mental health.

## Data Availability Statement

The datasets presented in this study can be found in online repositories. The names of the repository/repositories and accession number(s) can be found in the article/Supplementary Material.

## Ethics Statement

The studies involving human participants were reviewed and approved by ALSPAC Ethics and Law Committee. Written informed consent to participate in this study was provided by the participants' legal guardian/next of kin.

## Author Contributions

SS conceived the idea, analyses the data, and wrote the paper. SY, SA, KE, KK, IC, CD, SZ, and AN co-authored the paper and commented on drafts.

## Conflict of Interest

The authors declare that the research was conducted in the absence of any commercial or financial relationships that could be construed as a potential conflict of interest.
