# Supplementary material for: The Association Between Locus of Control and Psychopathology: A Cross-Cohort Comparison Between a UK (Avon Longitudinal Study of Parents and Children) and a Japanese (Tokyo Teen Cohort) Cohort
Source: Front Psychol. 2021 Apr 21;12:600941. doi: 10.3389/fpsyg.2021.600941 (PMC8096915; doi:10.3389/fpsyg.2021.600941)
Supplement: Supplementary file 1 [file Table_1.DOCX]

**Online Supplement**

**Supplement A: Details of confounding variables**

| **Variable** | **Questionnaire Response Categories** | **Collection Time point** | **Assessment Tool** | **Respondent** |
| --- | --- | --- | --- | --- |
| **ALSPAC** | | | | |
| Gender | M/F | Birth | Cohort specific questionnaire | Mother |
| Mother’s education level | CSE, Vocational, O level, A level, Degree | Birth | Cohort specific questionnaire | Mother |
| Home ownership status | Mortgaged, Owned, Council rented, Rented privately furnished, Rented privately unfurnished, Housing association rented, Other | Birth | Cohort specific questionnaire | Mother |
| Family Income Range per month £ | 75.90-806.65 | 33 and 47 months | Cohort specific questionnaire | Mother |
| Mother’s marital status | Never married, widowed, divorced, separated, 1^st^ marriage, marriage 2 or 3 | Birth | Cohort specific questionnaire | Mother |
| IQ Range | 75-151 | 8 years | WISC | Child |
| **TTC** | | | | |
| Gender | M/F | 10 years | Cohort specific questionnaire | Caregiver |
| Education level of mother or father | Elementary school, junior high school, senior high school, two-year college, professional school, university, graduate school | 10 years | Cohort specific questionnaire | Caregiver |
| Home ownership status | Owned, rented, salary housing (company housing, civil service housing etc). | 10 years | Cohort specific questionnaire | Caregiver |
| Household Income Yen over previous year. Range | 1000-11,000 | 10 years | Cohort specific questionnaire | Caregiver |
| Identity of partner | Biological father, biological mother, other | 10 years | Cohort specific questionnaire | Caregiver |
| IQ Range | 51-144 | 10 years | WISC | Child |

**Supplement B: Cross-cohort confounder comparison**

| **Variable** | **ALSPAC mean (SD) n (%)** | **TTC mean (SD) n (%)** |
| --- | --- | --- |
| Gender F | 49.9% | 46.9% |
| Highest parental education (Degree vs other) | 12.8% | 71.2% |
| Home ownership status (Owned/mortgaged vs other) | 72.3% | 69.9% |
| Family Income (Upper 30% vs lower 70%) | NA | NA |
| Mother’s marital status/identity of partner (biological father vs other) | 74.2% | 98.4% |
| IQ | 102.3 (SD21.6) | 107.71 (SD 14.12) |

**Supplement C: Cross cohort comparison of severity of PLE at 14 years**

| PLE category n (%) | TTC @14 | ALSPAC @14 years | ALSPAC @ 16 years | ALSPAC @22 years |
| --- | --- | --- | --- | --- |
| None | 73.7% | 58.2% | 66.8% | 79.3% |
| Maybe | 15.8% | 29.9% | 21.3% | 14.7% |
| Definite but not distressing or frequent | 1.4% | 5.0% | 5.7% | 3.1% |
| Definite and distressing OR frequent | 5.0% | 5.3% | 5.1% | 2.2% |
| Definite and distressing AND frequent | 4.1% | 1.5% | 1.2% | 0.8% |

**Supplement D: Cross cohort missing data comparison**

| **Variable** | **N % missing ALSPAC** | **N % missing TTC** |
| --- | --- | --- |
| **PLE @ 22 years** | **10746 (76.8%)** |  |
| **PLEs @ 16 years** | **9104 (65.1%)** |  |
| **PLEs @ 14 years** | **8270 (59.1%)** | **1109 (35.0%)** |
| **PLEs @ 13 years** | **7230 (51.7%)** |  |
| **PLEs @ 12 years** |  | **632 (19.9%)** |
| **PLEs @ 11 years** | **6815 (48.7%)** |  |
| **DSs @ 22 years** | **12005 (85.8%)** |  |
| **DSs @ 16 years** | **10381 (74.2%)** |  |
| **DSs @ 14 years** | **11967 (85.6%)** | **1101 (34.7%)** |
| **DSs @ 13 years** | **10906 (78.0%)** |  |
| **DSs @ 12 years** |  | **692 (21.8%)** |
| **DSs @ 11 years** | **9438 (67.5%)** |  |
| **LoC @ 16 years** | **10,382 (74.2%)** |  |
| **LoC @ 10 years** |  | **188 (5.9%)** |
| **LoC @ 8** | **9053 (64.7%)** |  |

**Supplement E: Univariable estimates for the association between externality and PLE**

| Timepoint of PLE data collection years | 11 |  |  | 12 |  |  | 13 |  |  | 14 |  |  | 16 |  |  | 22 |  |  |
| --- | --- | --- | --- | --- | --- | --- | --- | --- | --- | --- | --- | --- | --- | --- | --- | --- | --- | --- |
|  | OR | 95% CI | p | OR | 95% CI | p | OR | 95% CI | p | OR | 95% CI | p | OR | 95% CI | p | OR | 95% CI | p |
| **ALSPAC** | | | | | | | | | | | | | | | | | | |
| Sample size n | 4190 |  |  |  |  |  | 3995 |  |  | 3448 |  |  | 3019 |  |  | 2051/1955 |  |  |
| Externality @ 8 | 1.05 | 1.02, 1.09 | 0.002 |  |  |  | 1.09 | 1.04, 1.13 | ≤0.0001 | 1.06 | 1.01, 1.11 | 0.018 | 1.06 | 1.01, 1.12 | 0.025 | 1.04 | 0.96, 1.13 | 0.331 |
| Externality @ 16 |  |  |  |  |  |  |  |  |  |  |  |  |  |  |  | 1.27 | 1.17, 1.39 | ≤0.0001 |
| **TTC** | | | | | | | | | | | | | | | | | | |
| Sample size n |  |  |  | 2309 |  |  |  |  |  | 1878 |  |  |  |  |  |  |  |  |
| Externality @ 10 |  |  |  | 1.12 | 1.06, 1.18 | ≤0.0001 |  |  |  | 1.10 | 1.03, 1.17 | 0.004 |  |  |  |  |  |  |

**Supplement F: Univariable estimates for the association between externality and DSs**

| Timepoint of sMFQ data collection (years) | 11 |  |  | 12 |  |  | 13 |  |  | 14 |  |  | 16 |  |  | 22 |  |  |
| --- | --- | --- | --- | --- | --- | --- | --- | --- | --- | --- | --- | --- | --- | --- | --- | --- | --- | --- |
|  | β | 95% CI | p | β | 95% CI | p | β | 95% CI | p | β | 95% CI | p | β | 95% CI | p | β | 95% CI | p |
| **ALSPAC** | | | | | | | | | | | | | | | | | | |
| Sample size n | 3810 |  |  |  |  |  | 2725 |  |  | 2150 |  |  | 3156 |  |  | 1996/1900 |  |  |
| Externality @ 8 | 0.12 | 0.06, 0.19 | ≤0.0001 |  |  |  | 0.15 | 0.06, 0.24 | 0.001 | 0.19 | 0.08, 0.31 | 0.001 | 0.15 | 0.05, 0.24 | 0.002 | 0.11 | -0.004, 0.22 | 0.059 |
| Externality @ 16 |  |  |  |  |  |  |  |  |  |  |  |  |  |  |  | 0.59 | 0.48, 0.71 | ≤0.0001 |
| **TTC** | | | | | | | | | | | | | | | | | | |
| Sample size n |  |  |  | 2250 |  |  |  |  |  | 1888 |  |  |  |  |  |  |  |  |
| Externality @ 10 |  |  |  | 0.34 | 0.25, 0.44 | ≤0.0001 |  |  |  | 0.15 | 0.04, 0.26 | 0.010 |  |  |  |  |  |  |
